# Intermittent hypoxic resistance training: does it provide added benefit?

**DOI:** 10.3389/fphys.2014.00397

**Published:** 2014-10-13

**Authors:** Brendan R. Scott, Katie M. Slattery, Ben J. Dascombe

**Affiliations:** ^1^Applied Sports Science and Exercise Testing Laboratory, Faculty of Science and Information Technology, University of NewcastleOurimbah, NSW, Australia; ^2^New South Wales Institute of SportSydney, NSW, Australia

**Keywords:** muscle, hypertrophy, strength, metabolic stress, training adaptation

## Introduction

Methods to enhance the adaptive responses to resistance training are of great interest to clinical and athletic populations alike. Altering the muscular environment by restricting oxygen availability during resistance exercise has been shown to induce favorable physiological adaptations. An acute hypoxic stimulus during exercise essentially increases reliance on anaerobic pathways, augmenting metabolic stress responses, and subsequent hypertrophic processes (Scott et al., 2014). Hypoxic strategies during resistance exercise were originally investigated using blood flow restriction (BFR) methods (Takarada et al., 2000), whereby a cuff is applied proximally to a limb to partially limit arterial inflow while occluding venous outflow from the working muscles. Another method that has been investigated more recently is performing resistance exercise in systemic hypoxia, by means of participants breathing a hypoxic air mixture.

The addition of systemic hypoxia to resistance training has previously resulted in significantly enhanced hypertrophic and strength responses to both low-load (20% 1-repetition maximum; 1RM) (Manimmanakorn et al., 2013a,b) and moderate-load (70% 1RM) (Nishimura et al., 2010) resistance training. While research into intermittent hypoxic resistance training (IHRT) is in its infancy, some studies have reported conflicting results, which is likely due to differing research methodologies. In a recent review, it has been suggested that many of the potential mechanisms underpinning muscle adaptations to BFR training and IHRT are linked to the muscular oxygenation status and degree of metabolic stress associated with exercise (Scott et al., 2014). The purpose of this paper is to briefly summarize the adaptive responses that have been reported following both low- and moderate-load IHRT and to highlight key areas of concern for IHRT methodology, including the level of hypoxia used and the degree of metabolic stress imposed during exercise.

## Findings from IHRT studies

To date, six separate investigations have examined the impact of IHRT on hypertrophic and strength responses, resulting in seven published papers comparing adaptive responses following IHRT to a normoxic control group (Friedmann et al., 2003; Nishimura et al., 2010; Manimmanakorn et al., 2013a,b; Ho et al., 2014b; Kon et al., 2014; Kurobe et al., 2014). Research using low-load resistance training (20% 1RM) combined with moderate hypoxia (fraction of inspired oxygen [F_I_O_2_] adjusted to maintain arterial oxygen saturation at 80%) and very brief inter-set rest periods (30 s) has reported greater hypertrophic and strength responses following IHRT compared to work-matched normoxic training (Manimmanakorn et al., 2013a,b). However, another study using similar exercise loads (30% 1RM), yet longer inter-set rest periods (60 s) and a greater hypoxic stress (F_I_O_2_ = 12%) has observed no additive benefits of IHRT (Friedmann et al., 2003). While conflicting, these findings may indicate that for IHRT using low-loads, both the duration of inter-set rest periods and the level of hypoxia affect the adaptive responses.

Nishimura et al. (2010) used moderate-load resistance training (70% 1RM) combined with moderate-level hypoxia (F_I_O_2_ = 16%) and a relatively brief inter-set rest period (60 s), demonstrating enhanced hypertrophic, and strength responses following IHRT compared to the equivalent training in normoxia. However, research using similar exercise loads (70% 1RM or 10RM) and levels of hypoxia (F_I_O_2_ = 14.4–15%) in conjunction with longer inter-set rest periods (90–120 s) has not found additive hypertrophic or strength benefits for IHRT (Ho et al., 2014b; Kon et al., 2014). Furthermore, a study that employed moderate-load exercise (10RM) and brief inter-set rest (60 s), but a high-level of hypoxia (F_I_O_2_ = 12.7%) noted significantly greater hypertrophic, but not strength adaptations, following IHRT compared to normoxic training (Kurobe et al., 2014). When considering the available evidence, it appears that moderate hypoxia in conjunction with relatively brief inter-set rest periods (and subsequently increased metabolic stress) are paramount in enhancing muscular development via IHRT.

## Level of hypoxia

The hypoxic stimulus used in previous IHRT investigations has ranged from F_I_O_2_ = 12–16%, or investigators have adjusted the hypoxic stimulus to maintain arterial oxygen saturation at 80%. Importantly, no two investigations have employed the same hypoxic stimulus, which has led to differing physiological responses, and difficulty in directly comparing the findings of each study. It has been established that the level of hypoxia or altitude an individual is exposed to has a dose-response relationship on subsequent markers of endurance performance (Chapman et al., 2014), and it is logical that a similar relationship may exist for muscular development following IHRT.

It is possible that for enhanced muscle hypertrophy and strength development from IHRT, the level of hypoxia may follow a hermetic relationship, meaning that some beneficial acute responses to hypoxia may be attenuated if the level of hypoxia is too high. Muscle function during IHRT using high-levels of hypoxia could be impacted by the presence of a “central governor,” which was popularized by Noakes et al. (2001). This theory postulates that the degree of motor unit recruitment by the central nervous system is determined by the brain's need to protect itself and the body, ensuring survival, and maintenance of integrity during and following exercise (Millet et al., 2009). As such, a hypoxia-mediated reduction in central drive may occur above a certain hypoxic threshold. Amann et al. (2006) have hypothesized that oxygen supply affects the regulation of motor output to ensure that muscular fatigue does not exceed a critical threshold.

It has been previously reported that exercising in systemic hypoxia can induce changes in cerebral oxygenation, which in turn may limit incremental exercise performance (Subudhi et al., 2007). Altered cerebral oxygenation may play a regulatory role during IHRT using high-level hypoxia, effectively attenuating any increases in muscle activation that may occur using moderate-level hypoxia. This cerebral mechanism is unlikely to be present during BFR exercise, where the hypoxic environment is localized to the limb being trained and significant elevations in muscle activation are regularly reported (Takarada et al., 2000; Yasuda et al., 2009). This is a probable difference between these two resistance training methods. Although further research is needed to examine the dose-response relationship between the level of hypoxia during IHRT and subsequent muscle activation, current evidence suggests that beneficial muscular adaptations are only possible when moderate-level hypoxia is employed in conjunction with brief inter-set rest periods (Nishimura et al., 2010; Manimmanakorn et al., 2013a,b).

## Metabolic stress

The degree of metabolic stress associated with resistance training has been proposed as an important regulator of subsequent adaptive muscular responses (Schoenfeld, 2013). Intramuscular hypoxia during resistance exercise likely increases the reliance on anaerobic metabolism (Kawada, 2005), which accelerates the production of metabolites. Indeed, researchers have previously demonstrated that IHRT (F_I_O_2_ = 13%) using both low-loads (5 sets of 14 repetitions at 50% 1RM with 60 s inter-set rest) (Kon et al., 2012) and moderate-loads (5 sets of 10 repetitions at 70% 1RM with 60 s inter-set rest) (Kon et al., 2010) can increase metabolic stress, measured via blood lactate concentration, when compared to the equivalent exercise in normoxia. As such, increased metabolic stress may be a primary mechanism underpinning augmented muscular responses to hypoxic resistance training methods.

Schoenfeld (2013) proposed hypertrophic adaptations to resistance exercise may be mediated by metabolic stress via enhanced muscle activation, up-regulated endocrine responses, greater production of local myokines and reactive oxygen species, and cellular swelling. If hypoxia-mediated increases in metabolic stress are in fact an underlying mechanism for adaptation to IHRT, it is important to understand how best to utilize hypoxic stimuli to augment metabolic stress. If this is not considered during IHRT program design, it is likely that mechanisms downstream from the metabolic stress will not be further enhanced, and no additive benefits will be observed for IHRT. One key area factor that has received limited research attention is the duration of inter-set recovery periods. As highlighted by Bird et al. (2005), the length of inter-set rest periods not only determines the degree of adenosine triphosphate-PCr energy recovery, but also the extent to which blood lactate concentrations are elevated. Importantly, IHRT is vastly different to BFR methods in this regard, given that venous outflow is occluded by the BFR stimulus and metabolites therefore cannot be re-distributed away from the exercising limb. However, venous outflow is maintained during IHRT, meaning that metabolites can enter circulation and be distributed to other parts of the body. Logic therefore dictates that for augmented metabolic stress during IHRT, inter-set rest periods should be short enough to ensure that a hypoxia-mediated metabolic stress is still present within the muscles during each subsequent set.

A mechanism by which hypoxia may alter energetic metabolism during resistance exercise may by slowing the rate of phosphocreatine (PCr) recovery between sets. Resynthesis of PCr occurs primarily by oxidative processes, and is therefore sensitive to manipulations of oxygen availability (Haseler et al., 1999). It is possible that relatively brief inter-set rest periods will result in subsequent sets beginning with a lower PCr concentration when training in hypoxia, placing greater stress on anaerobic glycolysis, and consequently increasing the accumulation of metabolites. This may account for the findings of no added hypertrophic or strength benefits following IHRT in previous research that has employed inter-set rest periods of 90–120 s (Ho et al., 2014b; Kon et al., 2014). Indeed, Ho et al. (2014a) have reported no differences in blood lactate concentrations between hypoxic and normoxic IHRT groups following 5 sets of 15 repetitions of squats (30% 1RM) with 90 s inter-set rest. Therefore, it is likely that inter-set rest periods of 90 s or longer are sufficient to attenuate any hypoxia-mediated rise in metabolic stress, limiting the potential anabolic effects of hypoxia.

While some research has previously demonstrated increased metabolic stress following IHRT (Kon et al., 2010, 2012), conflicting data have been recently reported (Kurobe et al., 2014; Ho et al., 2014a). Kurobe et al. (2014) observed no differences between hypoxic and normoxic training groups performing 3 sets of 10 repetitions of elbow extensions (10RM) with 60 s inter-set rest. These conflicting results may be partly explained by the differences in exercise volume, as the studies by Kon et al. (2010, 2012) both employed 5 sets of two exercises, whereas Kurobe et al. (2014) employed a single exercise for 3 sets. In addition, the study by Ho et al. (2014a) used very long inter-set recovery periods, especially considering the low exercise loads prescribed, which likely attenuated any hypoxia-mediated increase in metabolic stress. It is possible that hypoxic conditions facilitate only a small increase in metabolic stress, which requires more than 3 sets with short rest intervals between to provide a significant accumulated effect. However, this explanation remains speculative and requires further research.

## Conclusions

When considering the current research into IHRT, it is clear that methodological inconsistencies between studies may have caused the conflicting results. While there is currently a limited body of research examining IHRT, it appears that adaptive responses may be influenced by the level of hypoxia and the inter-set rest periods (and subsequent alterations in metabolic stress) used. We propose that for IHRT to elicit greater muscular adaptations than the equivalent training in normoxia, exercise protocols should be designed to provide a substantial metabolic stimulus, with particular care being taken to implement relatively brief rest periods between each set. However, it should also be acknowledged that a range of other factors could influence adaptive responses to IHRT. For example, neuroplasticity may be altered by long periods of intermittent hypoxic exposures, and could therefore play a role in adaptation to extended IHRT programs. Nonetheless, the purpose of this paper is to provoke thought amongst scientists regarding how resistance training program design may be manipulated in conjunction with systemic hypoxia to enhance adaptive responses. Given the available evidence, we suggest that inter-set rest periods should be very brief for low-load exercise (~ 20–30% 1RM; ~30 s) and brief for moderate-load exercise (~70% 1RM; ~60 s). Furthermore, it may also be important to ensure that a moderate, rather than high, level of hypoxia is used (F_I_O_2_ = ~14–16%).

### Conflict of interest statement

The authors declare that the research was conducted in the absence of any commercial or financial relationships that could be construed as a potential conflict of interest.
